# Antihyperlipidemic and Antioxidant Capacities, Nutritional Analysis and UHPLC-PDA-MS Characterization of Cocona Fruits (*Solanum sessiliflorum* Dunal) from the Peruvian Amazon

**DOI:** 10.3390/antiox10101566

**Published:** 2021-09-30

**Authors:** Gabriel Vargas-Arana, Claudia Merino-Zegarra, Marcos Riquelme-Penaherrera, Luis Nonato-Ramirez, Henry Delgado-Wong, Mariano Walter Pertino, Claudio Parra, Mario J. Simirgiotis

**Affiliations:** 1 Laboratorio de Química de Productos Naturales, Instituto de Investigaciones de la Amazonía Peruana, Av. Abelardo Quiñones km 2.5, Iquitos 16001, Peru; cmerino@iiap.gob.pe; 2 Facultad de Farmacia y Bioquímica, Universidad Nacional de la Amazonía Peruana, Iquitos 16001, Peru; mriquelmepenaherrera@diresaloreto.gob.pe (M.R.-P.); luis.nonato@unapiquitos.edu.pe (L.N.-R.); henry.delgado@unapiquitos.edu.pe (H.D.-W.); 3 Laboratorio de Química de Productos Naturales, Instituto de Química de Recursos Naturales, Universidad de Talca, Casilla 747, Talca 3460000, Chile; mwalter@utalca.cl; 4 Laboratorio de Química Orgánica y Productos Naturales, Facultad de Ciencias Agronómicas, Universidad de Tarapacá, Av. General Velásquez 1775, Arica 1000000, Chile; cparra@uta.cl; 5 Instituto de Farmacia, Facultad de Ciencias, Universidad Austral de Chile, Valdivia 509000, Chile

**Keywords:** antioxidant activity, UHPLC-PDA-ESI-OT-MS, Solanaceae, nutritional values, phenolics, antihyperlipidemic

## Abstract

Cocona fruits are a popular food and medicinal fruit used mainly in the Amazon and several countries of South America for the preparation of several food products such as drinks, jams and milk shakes. In this study five ecotypes of cocona native to Peru have been studied regarding their nutritional and antioxidants values plus antihyperlipidemic activities. Seventy bioactive compounds have been detected in Peruvian cocona ecotypes including several phenolic acids, aminoacids and flavonoids; of those six were spermidines, (peaks 1, 2, 25, 26, 38 and 39), thirteen were aminoacids, (peaks 3–9, 11–13, 16, 17, 22–24), eighteen flavonoids (peaks 28, 30–32 45,46, 48–53 56, 57, 61 and 64–66), twelve were phenolics (peaks 19, 21, 27, 29, 34, 35, 36, 42, 43, 44, 54, and 59), two carotenoids, (peak 62 and 63), eight were lipid derivatives (peaks 37, 55, 58, 60 and 67–70), one sugar (peak 47), four terpenes (peaks 33, 40, 41 and 47), two amides, (peaks 10 and 18), one aldehyde, (peak 15), and three saturated organic acids, (peaks 4, 5 and 20). Hypercholesterolemic rats administered with pulp of the ecotypes CTR and SRN9 showed the lowest cholesterol and triglyceride levels after treatment (126.74 ± 6.63; 102.11 ± 9.47; 58.16 ± 6.64; 61.05 ± 4.00 mg/dL, for cholesterol, triglycerides, high-density lipoprotein and low-density lipoprotein respectively, for the group treated with SRN9 pulp, and 130.09 ± 8.55; 108.51 ± 10.04; 57.30 ± 5.72; and 65.41 ± 7.68 mg/dL, for cholesterol, triglycerides, HDL and LDL lipoproteins respectively for the group treated with CTR pulp). The ecotypes proved to be good sources of natural antioxidants and their consumption represent an alternative for the prevention of atherosclerosis.

## 1. Introduction

**Figure 1:**
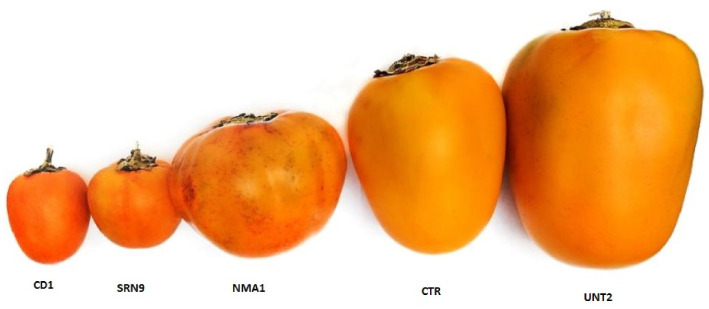
Pictures of cocona fruits NMA1, SRN9, CD1, CTR, UNT2 ecotypes.

In recent decades, global interest has increased in search of the chemical composition and biological activities of natural sources since many of the compounds present in biological sources such as local plants and marine organisms are important for the protection of human health. The fruit of cocona (*Solanum sessiliflorum* Dunal; Solanaceae) are native of the Amazonian tropic. Just like other plants in the genus Solanum, they exhibit a morphological diversity corresponding to the variability in habitat and ecology changes, as well as with the process of domestication of the species; However, little is known about its chemical composition and the implications of the terroir and climate in its morphology and nutrient and health beneficial properties. The common name of the fruit in Spanish or Portuguese speaking countries is cocona, topiro or cubiu, and is known as “Orinoco apple” and “peach tomato” in English speaking countries. It is an endemic species to Amazon cultivated by natives and settlers in agroforestry arrangements and chagras in their settlement sites which improves it on a traditional food from this area. The pulp is the edible part of the fruit and is known for its refreshing flavor to produce refreshments, milk shakes, jams, and jellies and for the medicinal properties, including amelioration of itching produced by insect bites, elimination of parasites, it is also use as topical to heal burns and for the control of cholesterol, diabetes, and uric acid. The studies carried out on the chemistry of the species are few and no complete, mainly limited to the report of some phenolic compounds and volatile metabolites which cannot depict the differentiation in the variations in the chemical composition between the different morphotypes of the fruit which is very important to give added values to the different products and ecotypes. This study aimed to differentiate five varieties of cocona (Figure 1) with the use of UHPLC coupled to high resolution mass spectrometry (UHPLC-PDA-ESI-OT-MS) grown in Peru, called oval, small round, large round big oval round and big square morphotypes, by means of the metabolite fingerprinting of the secondary metabolites present in mature fruits of the species. These adaptive morphotypes of the fruit depends directly on various biological factors and has been diversifying during the evolution and natural selection and can have the ability to produce different biologically important metabolites. Several carotenoids, alkaloids, organic acids, phenolic acids, flavonoid glycosides, coumarins, tannins, and volatile and fixed acids were reported to occur in fresh cocona fruits from Brazil [1,2] and recently, caffeoyl quinic acid was reported as the main important phenolic in the fruits [3], whereas determination of volatile organic compounds (VOCs) was done by HS-SPME/GC-MS in some ecotypes from Brazil [4], besides, extracts of those Brazilian ecotypes presented high concentrations of caffeic and gallic acids, beta-carotene, catechin, quercetin, and rutin and showed low density lipoproteins oxidation; cytotoxic and antiproliferative effect on breast (MCF-7) and colorectal (HT-29) cancer cell lines [5]. The purpose of this work is to contribute to the full phytochemical study of the Peruvian ecotype species through UHPLC-PDA-ESI-OT-MS for full untargeted metabolomic analyses to promote in fruit growers and fruit processors in the Peruvian Amazon the adoption of strategies for the sustainable use of the more promising ones based on its phenolic content and intrinsic health related properties, such as antihyperlipidemic capacities of the studied five ecotypes of this highly consumed fruit from Peru. Finally, proximal composition and mineral contents, plus the antioxidant activities thorough different methods and total carotene and phenolic contents of all ecotype pulps were tested and compared. The UHPLC full MS and PDA fingerprint analysis, was also performed for the ecotypes. 

## 2. Materials and Methods

### 2.1. Chemicals

Ultra-pure water (<5 µg/L TOC, (total organic carbon)) was obtained from a water purification system Arium 126 61316-RO, plus an Arium 611 UV unit (Sartorius, Goettingen, Germany). Methanol (HPLC grade) and formic acid (puriss. p.a. for mass spectrometry) from J. T. Baker (Phillipsburg, NJ, USA) were obtained. Dichloromethane (HPLC grade) were from Merck (Santiago, Chile). Commercial Folin–Ciocalteu (FC) reagent, 2,2-diphenyl-1-picrylhydrazyl (DPPH), ferric chloride hexahydrate, 2,4,6-tris(2-pyridyl)-s-triazine, trolox, quercetin, gallic acid, atorvastatin, Triton WR-1339 and DMSO were purchased from Sigma-Aldrich Chem. Co. (St Louis, MO, USA).

### 2.2. Plant Material and Sample Treatment

The study was carried out with five ecotypes of *Solanum sessiliflorum*, whose seeds were obtained from the ex situ conservation gene bank of the Peruvian Amazon Research Institute, cultivated in the Research and Production Center of Tulumayo of the National Agrarian University of the Jungle (09°06′20″ S and 75°54′15″ W, 565 meters altitude) Huanuco region, Peru. The ecotypes were selected based on their morphotypic difference, which were: CTR, collected in cascas, Lambayeque region, SRN9, collected in Supte Black river, Huanuco region, UNT2, collected in Padre Abad, Ucayali region, NMA1, collected in Leoncio Prado, Huanuco region, and CD1, collected in Bagua, Amazonas region. The selected fruits were washed, brushed, disinfected using a 200-ppm sodium hypochlorite solution for 30 min and rinsed with water. The epicarp was then removed. Then thermal bleaching was carried out at 90 °C for 20 min. The pulp was then extracted in a pulping machine removing the husks and seeds. Finally, the extracted pulp was lyophilized. The lyophilized pulp was packed in plastic bags and stored in a freezer at −20 °C until use (for some days only). Lyophilized pulp (2.0 g) was extracted two times in 5 mL of methanol/water (MeOH/H_2_O) solutions (80/20, *v*/*v*) under sonication (130 kHz, 10 min) at room temperature. Then, samples were centrifuged (5000 rpm, 15 min, 5 °C) and the supernatants were combined after filtration and stored at −20 °C before analyses.

### 2.3. UHPLC-PDA-ESI-OT-MS Instrument

UHPLC-PDA-ESI-OT-MS analysis was carried out as described by [6], with some modifications, Briefly, a Thermo Scientific Dionex Ultimate 3000 UHPLC system hyphenated with a Thermo Q exactive plus machine was used. For the analysis 5 mg of the extract were dissolved in 2 mL of methanol, filtered (PTFE filter) and 10 µL were injected in the instrument, using an UHPLC C-18 column (Luna© Omega C-18 100 Å, Phenomenex 150 mm × 2.1 mm × 1.6 µm), operated at 25 °C. The detection wavelengths were 254, 280, 330 and 354 nm, and PDA was recorded from 200 to 800 nm for peak characterization. Mobile phases were 1% formic aqueous solution (A) and 1% formic acid in acetonitrile (B). The gradient program (time (min), % B) was: (0.00 min, 5% B); (1.500 min, 15% B); (1.5 min, 5% B); (35.00 min, 95% B); (36.00, 95% B); (38.00 min, 5% B); and 15 min for column equilibration before each injection. The flow rate was 0.300 mL min^−1^, and the injection volume was 10 µL. Standards and the pulp extract dissolved in methanol were kept at 10 °C during storage in the autosampler. Parameters for Full MS scan: Maximum IT: 80 ms, AGC target: 5 × 106 Resolution: 35,000 Range: 100–1500 *m*/*z*, Microscans: 1, Parameters MS2 Maximum IT: 100 ms AGC target: 1 × 106 Resolution: 17,500, Ionization source parameters: ESI (positive and negative) spray volt: 3.5/2.5 KV, Capillary temperature: 260 °C, Carrier gas: N2 (Sheath gas flow rate: 48, Sweep gas flow rate: 2) Gas heater temp: 280/280 °C, S-lens RF level: 100.

### 2.4. Antioxidant Activity Assays 

#### 2.4.1. DPPH Test

The DPPH• radical was assayed by the decolorization method [7]. Briefly, 9 μL of extract, (2 mg/mL), plus 341 μL of methanol DPPH solution (400 μM) were adjusted with the solvent methanol to an absorbance of 1.10 ± 0.02 at 517 nm. The mixture was homogenized using a vortex, allowed to react in the dark at room temperature for 20 min, after which time absorbance was measured at 517 nm in a Synergy HTX monochromator (Biotek, USA). The percentage of decoloration of the DPPH moiety was obtained by measuring the change in absorbance at 517 nm, the values obtained converted to percent inhibition of the DPPH moiety. The results are expressed in TEAC, that is, antioxidant activity equivalent to Trolox (μmol Trolox/g of lyophilized pulp). The synthetic antioxidant reference Trolox, at a concentration of 5–30 µM in 80% methanol solution, is tested under the same conditions.

#### 2.4.2. ABTS Method

The ABTS assay was performed by bleaching of the cationic radical ABTS^•+^ as described by [8]. For the preparation of the radical ABTS^•+^ 2.5 mL of the 7 mM ABTS solution, it was mixed with 2.5 mL of 2.45 μM sodium persulfate for 12 hours in the dark at 4 °C. Then, the resulting solution was diluted with absolute ethanol until an initial absorbance of approximately 0.70 ± 0.03 was obtained at 734 nm. The radical discoloration was initiated by adding 50 µL of the extract to 150 µL of the ABTS^•+^ solution. After 15 minutes of incubation at 25 °C, the absorbance was measured at 734 nm and compared with a calibration curve using Trolox as standard and ethanol as blank. Results were expressed as micromoles of Trolox equivalents per gram of dry sample (µmol TE/g).

### 2.5. Polyphenol (Folin-Ciocalteau) 

Total phenolic compounds (TPC) were analyzed based on [6] and [9]. To 12 μL of extract to be measured, 168 μL of the 1% Folin-Ciocalteu reagent (Merck, Santiago, Chile) were added to well of a microplate reader. The mixture could react for 5 min, then 120 μL of 10% sodium carbonate was added. The mixture was incubated at room temperature for 30 min in darkness. Absorbance was then taken at 765 nm using an UV-Visible multiplate reader (Synergy HTX, Biotek, Winooski, VT, USA). The obtained absorbance values were replaced in the equation of the standard curve of gallic acid (μmol/L). The content regarding phenolic compounds was then expressed as gallic acid milligrams per gram of dry weight (mg GAE/g extract).

### 2.6. Determination of Proximal Composition

Water content was determined by oven drying the sample up to a constant weight, the crude protein content by the Kjeldahl method (N × 6.25), the fiber content by gravimetric method after acidic hydrolysis of the samples, the total lipid extracted in a Soxhlet apparatus using petroleum ether as solvent, the ash content by incineration in a muffle furnace at 550 ± 15 °C. AOAC procedures were used in all determinations [10].

Total carbohydrates were calculated as difference: 100 − (g water + g protein + g fiber + g fat + g ash). Results were expressed in g per 100 g fresh weight (g/100 g fw).

### 2.7. Mineral Analysis 

For the mineral analysis [11], the fresh fruits pulps were dry ashed at 550 °C. The ash in each case was boiled with 10 ml of 20% hydrochloric acid in a beaker, and then filtered into a 100 mL standard flask and made up to 100 ml with distilled deionized water. Levels of minerals, sodium (Na), potassium (K), magnesium (Mg), manganese (Mn), copper (Cu), zinc (Zn), iron (Fe) and calcium (Ca) were determined from the resulting solution using atomic absorption spectroscopy (Varian AA240). The values obtained for each parameter are averages of three determinations for a given food sample.

### 2.8. Total Carotene Content

Total carotenoids were extracted with hexane, acetone and ethanol. The supernatant from the hexane phase was extracted, rich in carotenoids, and its absorbance at 450 nm was determined. The calculation of total carotenoids was performed by comparison with a calibration curve obtained with a certified β-carotene standard [12]. The results were expressed as μg β-carotene g^−1^ sample.

### 2.9. Induction of Hypercholesterolemia 

For experimental induction of hypercholesterolemia, male albino rats of the Wistar strain (150–200 g) were housed under conditions of controlled temperature (25 ± 2 °C) with a 12 h/12 h day-night cycle, during which time they had free access to food and water ad libitum. Animals were maintained per national guidelines and protocols of guide for the handling and care of laboratory of the National Institute of health. Hypercholesterolemia was induced experimentally in 12 h-fasted rats by a single intraperitoneal injection of Triton WR-1339 (300 mg/kg Body weight (b.wt.)) dissolved in 0.89% saline [13]. Forty-eight hours after administration of Triton WR-1339, rats exhibited elevated serum levels of total cholesterol and triglycerides; these rats were deemed to be hypercholesterolemic and use for further investigation. The experimental rats were divided into eight treatment groups, each comprising six rats. Group I. Control rats (not hypercholesterolemic and did not receive any treatment). Group II. Hypercholesterolemic rats that received only saline orally for three days. Group III. Hypercholesterolemic rats that received atorvastatin (10 mg/kg b.wt./day) in an aqueous suspension orally for three days. Group IV. Hypercholesterolemic rats that received the NMA1 lyophilized pulp ecotype (500 mg/ b.wt./day) in an aqueous suspension orally for 3 days. Group V. Hypercholesterolemic rats that received the CD1 lyophilized pulp ecotype (500 mg/b.wt./day) in an aqueous suspension orally for 3 days.

Group VI. Hypercholesterolemic rats that received the CTR lyophilized pulp ecotype (500 mg/b.wt./day) in an aqueous suspension orally for 3 days. Group VII. Hypercholesterolemic rats that received the SRN9 lyophilized pulp ecotype (500 mg/b.wt./day) in an aqueous suspension orally for 3 days. Group VIII. Hypercholesterolemic rats that received the UNT2 lyophilized pulp ecotype (500 mg/b.wt./day) in an aqueous suspension orally for 3 days. Saline, atorvastatin and NMA1, CD1, CTR, SRN9, UNT2 lyophilized pulp ecotypes were administered orally by gastric intubation once daily for 3 days. Blood samples were collected from all experimental rats on day 6 (3 days after start of treatment), and, subsequently, serum was separated for subsequent analysis of serum lipid profile parameters. Mean levels of total cholesterol, triglycerides, high-density lipoprotein (HDL) cholesterol and low-density lipoprotein (LDL) cholesterol were determined by standard kits (Stambio, Boerne, TX, USA) following the manufacturer’s instructions. The units being expressed as milligrams per deciliter (mg/dL).

### 2.10. Statistical Analysis

The statistical analysis was carried out using the originPro 9.1 software packages (Originlab Corporation, Northampton, MA, USA). The determination was repeated at least three times for each sample solution. The Tukey comparison test determined significant differences between means (*p* values ≤ 0.05 were regarded as significant).

## 3. Results and Discussion

### 3.1. Nutritional and Physicochemical Properties of 5 Cocona Ecotypes

Table 1 shows proximal composition such as the humidity, ashes, protein, lipids, carbohydrates, and fiber, while Table 2 shows the mineral contents of five ecotypes of cocona. Proximal composition of cocona fruits NMA1, SRN9, CD1, CTR, UNT2 ecotypes was performed. The results of physicochemical properties showed that the proximal composition and the caloric value of this fruit are similar to that showed for other species, but with a high fiber (from 1.08 to 1.93) and carbohydrate content (from 3.12–4.24). The edible portion of cocona ecotypes were analyzed for mineral content (Ca, Na, Mg, K, Cu, Mn, Zn, and Fe). The foods were generally high in K (570.83–2382.24 mg K/100 g edible portion) and low in sodium (3.25–6.87 mg Na/100 g edible portion). The five ecotypes had the highest contents in most of the elements, especially in calcium (17.85–70.07 mg Ca/100 g edible portion) and iron (52–71 mg Fe/100 g edible portion). 

### 3.2. Metabolite Profiling using UHPLC-PDA-ESI-OT-MS

**Figure 2:**
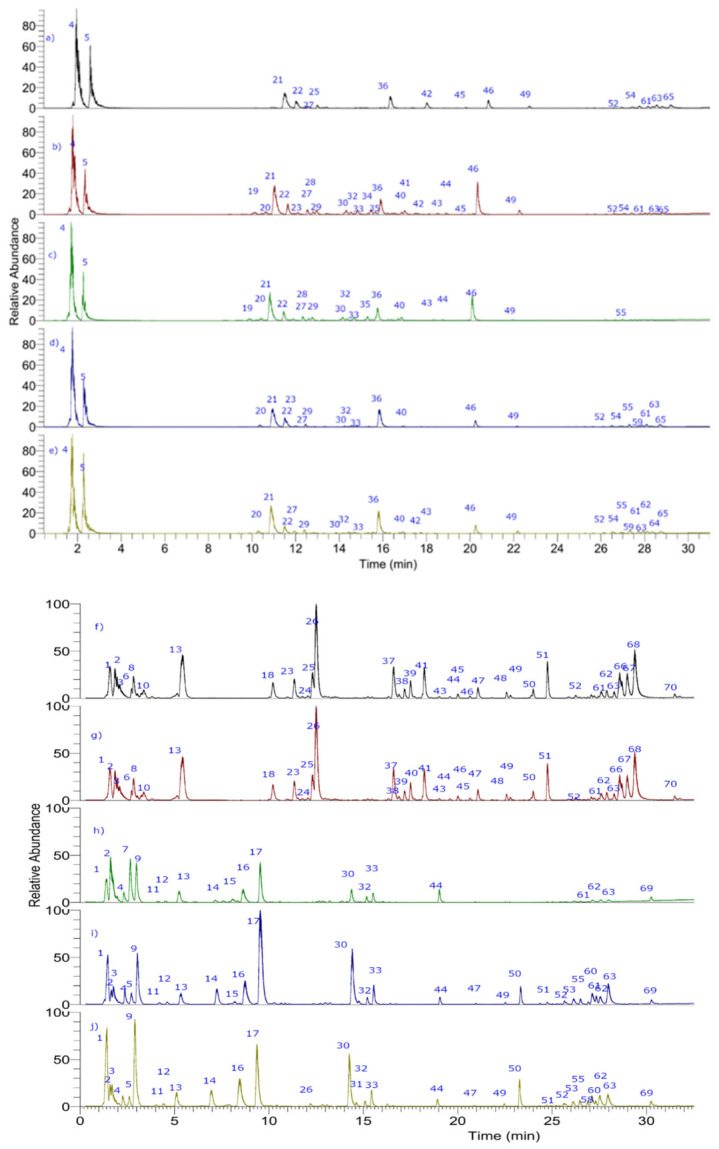
UHPLC-PDA-ESI-OT-MS chromatograms (TIC, total ion current) of cocona fruits NMA1, SRN9, CD1, CTR, UNT2 ecotypes: (**a**–**e**) positive mode and (**f**–**j**) negative mode.

**Figure 3:**
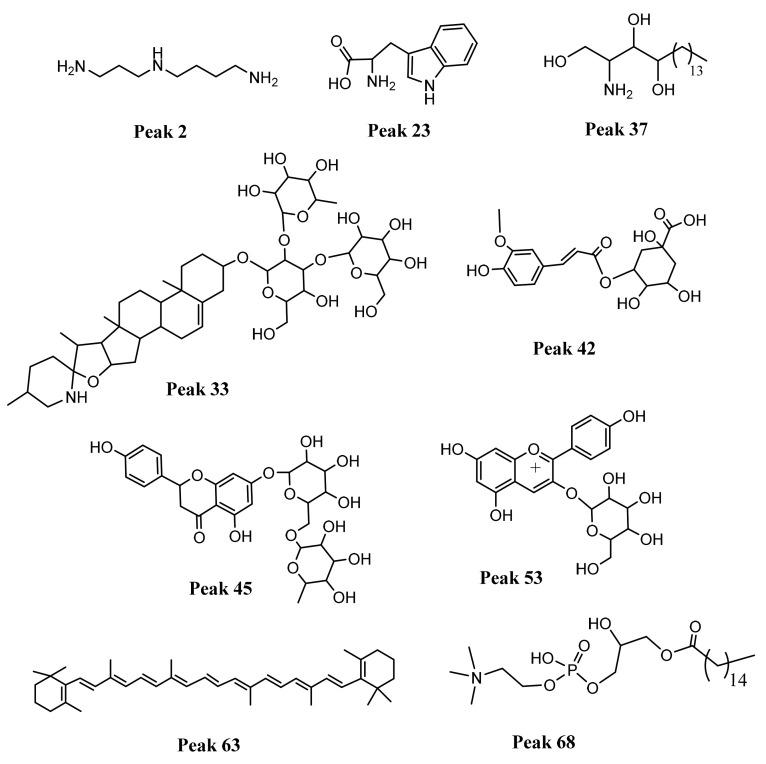
Structures of some representative compounds detected in cocona ecotypes: spermidine, peak 2, the aminoacid triptophan, peak 23, phytoesphingosine peak 37, spirosol-5-en-3-ol, 3-O-[rhamnosyl-glucosyl]-galactoside, peak 33, 3-O-feruloylquinic acid, peak 42, naringenin 7-O-rutinoside, peak 45, pelargonidin 3-O-glucoside, peak 53, B-carotene, peak 63 and 1-hexadecanoyl-sn-glycero-3-phosphocholine, peak 68.

The metabolite profiling was comprehensively performed by UHPLC-PDA-ESI-OT-MS, while Figure 2 shows the base peak UHPLC-mass chromatograms of ecotypes of cocona fruits and Table 3 shows the tentative identification of metabolites detected in the five ecotypes. Below are the detailed analyses, while Figure 3 show the structures of some representative compounds.

#### 3.2.1. Spermidines

Spermidine derivatives were previously reported by UHPLC MS by some of us in edible goji fruits [14]. Peak 1 with a [M+H] ^+^ ion at *m*/*z* 203.2229 is identified as spermine (C_10_H_26_N_4_), the parent ion producing some diagnostic daughter ions at *m*/*z* 129.1385, 112.1122, 84.0812 and 73.0813 and peak 2 as spermidine (C_7_H_19_N_3_), [14] peak 25 with a [M+H]^+^ ion at *m*/*z* 472.2441 as N-caffeoyl-N-(dihydrocaffeoyl) spermidine (C_25_H_33_N_3_O_6_) common components of potato tubers [15] and peak 26 as N-caffeoyl-N-(dihydrocaffeoyl)spermidine (C_26_H_37_N_3_O_6_) while peaks 38 and 39 as N,N″-Bis[3-(4-hydroxy-3-methoxyphenyl)propanoyl] spermidine (C_27_H_39_N_3_O_6_), and N,N,N-tris(dihydrocaffeoyl) spermidine (C_34_H_43_O_9_N_3_), Bioactive amines—such as spermidine and spermine-showed antioxidant activity in food through radical scavenging plus metal chelating properties, and were linked to reduced blood pressure and low incidence of cardiovascular diseases [16].

#### 3.2.2. Amines or Aminoacids

Several aminoacids were previously reported using orbitrap mass spectrometry by some of us [17]. Peak 3 was identified as histamine (C_5_H_9_N_3_), peak 6 as asparagine (C_4_H_8_N_2_O_3_), peak 7 with a [M+H]^+^ ion at *m*/*z* 175.1188 as the aminoacid arginine (C_6_H_14_N4O_2_), peak 9 as nicotinamide (C_5_H_7_NO_3_), Peak 10 as N-phenyl ethyl amide (C_8_H_10_N) and peak 11 with a protonated molecule at *m*/*z* 294.1544 and MS ions at *m*/*z* 276.1436, 258.1332, 230.1383, 212.1278 and 86.0968 as N-fructosyl isoleucine (C_12_H_23_NO_7_) and peak 12 as norleucine (C_6_H_13_NO_2_) and peak 13 as tyrosine (C_9_H_11_NO_3_), peak 14 as adenosine (C_10_H_13_N_5_O_4_) and peak 15 as phenylacetaldehyde (C_8_H_8_O), peak 16–18 and 22–23 as the aminoacids guanosine, phenylalanine, panthotenic acid and tryptophan, respectively. Peak 24 with a protonated molecule at *m*/*z* 144.0807 [M-H_2_O+H] as tryptophol while peak 18 as aminobutyl benzamide (C_11_H_16_N_2_O). Peak 55 was identified as 1-hexadecanoyl-sn-glycero-3-phosphoethanolamine (C_21_H_44_NO_7_P).

#### 3.2.3. Fatty Acids and Derivatives

Some oxylipins or unsaturated fatty acids were previously reported in edible fruits by some of us using TOF mass spectrometry [18]. In this study peak 37 with a pseudomolecular ion at *m*/*z* 318.3000, and daughter ions at *m*/*z* 318.2995, 300.2890, 282.2785, 270.2785, 60.0450, was identified as phytosphingosine (C_18_H_39_NO_3_) [19], and peak 54 as 1-(9Z,12Z-octadecadienoyl)-glycero-3-phospho-(1′-myo-inositol) (C_27_H_49_O_12_P), peak 58 as 1-hexadecanoyl-sn-glycero-3-phospho-(1′-myo-inositol) (C_25_H_49_O_12_P), peaks 66–70 as 1-(9Z,12Z-octadecadienoyl)-glycero-3-phospho-(1′-myo-inositol) (C_27_H_49_O_12_P), 1-(9Z-octadecenoyl)-sn-glycero-3-phosphoethanolamine (C_23_H_46_NO_7_P), 1-hexadecanoyl-sn-glycero-3-phosphocholine (C_24_H_50_NO_7_P), 1-(9Z-Octadecenoyl)-sn-glycero-3-phosphocholine (C_26_H_52_NO_7_P), and 1-Oleoyl-2-palmitoyl-sn-glycero-3-phosphocholine, (C_42_H_82_NO_8_P), respectively, and finally, peak 60 was identified as 1-(9Z-octadecenoyl)-sn-glycero-3-phospho-(1′-myo-inositol) (C_27_H_51_O_12_P).

#### 3.2.4. Phenolic Acids

Peak 19 was tentatively identified as chlorogenic acid (C_8_H_15_O_4_) and peak 20 as quinic acid, Peak 21 as 3-O-diglucosyl-4-methoxy-3-hydroxybenzoic acid and peak 27 as 3-O-diglucosyl-4-methoxy-3-hydroxybenzoic acid (C_20_H_28_O_14_), peak 29 as apiosyl-glucosyl-hydroxybenzoate (C_18_H_24_O_12_), peak 34 as 1-*O*-Sinapoyl-glucoside (C_17_H_22_O_10_), and peak 43 as 2-*O*-sinapoyl-glucoside (C_17_H_22_O_10_) [20], peak 35 as protocatechuic acid 5-O-apiofuranosyl-glucopyranoside (C_19_H_26_O_13_), peak 36 as 4-O-(3′-O-glucopyranosyl)-caffeoyl quinic acid (C_22_H_28_O_14_), peak 42 as 3-O-feruloylquinic acid (C_17_H_20_O_9_) [21], and peak 44 as syringaresinol 4-gentiobioside (C_34_H_46_O_18_), peak 53 as naringenin-7-O-glucoside (C_21_H_22_O_10_), [22] and peak 54 as methyl chlorogenate (C_17_H_20_O_9_) [23], finally, peak 59 was assigned as syringaresinol-glucoside (C_28_H_36_O_13_), and peak 65 as phloretin (C_15_H_14_O_5_). Several acyl quinic acids such as ferulic, sinapic and chlorogenic acid and derivatives showed different interesting bioactivities such as anti-inflammatory, vasorelaxant in mice aorta, platelet activation in human blood samples neuroprotective effects among others [24], while the glycoside derivative of the lignan syringaresinol enhanced ß-endorphin levels in rat plasma, [25] while syringaresinol aglycone was cytotoxic against human breast and lung cancer cells [26].

#### 3.2.5. Flavonoids

Peak 28 with was assigned as the flavonol glycoside rutin (C_27_H_30_O_16_) [27] with diagnostic ions at *m*/*z* 463.0920, 343.0465, 300.0280, 271.0252, 178.9982, and 151.0031, while peak 30 was assigned the structure naringenin-5,7-di-O-glucoside [28], (flavanone, Uv max 280 nm, C_27_H_31_O_15_^−^) and peak 31 as genistein 5-O-glucoside (isoflavone, UV max 280 nm, C_21_H_20_O_10_) and peak 32 as the flavanol isoquercitrin (C_21_H_20_O_12_) [27]. In the same way, peak 45 was identified as naringenin 7-O-rutinoside or neohesperidoside (C_27_H_31_O_14_^−^) [29], and peak 46 as quercetin 3-galactoside (C_12_H_19_O_5_^−^) Peak 48 was assigned to the isoflavone biochanin A 7-O-rutinoside (C_28_H_32_O_14_) [30], peaks 56 and 57 as the flavanones naringenin-5-O-glucoside (C_21_H_22_O_10_) and eriodictyol-7-O-glucoside (C_21_H_22_O_10_), [31] respectively. Peak 61 with a ion at *m*/*z* 579.2084 as the flavonol quercetin 3-O-malonylglucoside (C_24_H_22_O_15_) [32], while peaks 64 was assigned as naringenin, respectively. Some flavonoids showed antioxidant properties and inhibition of enzymes such as xanthine oxidase and others important such as anti-inflammatory activities [33,34,35].

#### 3.2.6. Terpenes

Peak 33 showing peaks at *m*/*z* 884.4987 and 928.4909 [M+FA-H], was tentatively assigned as the derivative of the terpene spirosol-5-en-3-ol,[36] 3-O-[rhamnosyl- glucosyl-galactoside] (C_45_H_73_NO_16_), and peak 40 as spirosol-5-en-3-ol, O-[rhamnosyl-[xylosyl- rhamnosyl-galactoside and peak 41 as cholest-5-ene-3,16,22,26-tetrol, 3-O-[Rhamnosyl- -rhamnosyl-glucoside], 26-O-glucoside (C_51_H_86_O_22_), and finally peak 47 as spirosol-5-en-3-ol, 3-O-[rhamnosyl- [rhamnosyl-glucoside] (C_45_H_73_NO_15_).

#### 3.2.7. Cyanidins

Peak 52 with a positive ion at *m*/*z* 595.1656 was assigned to the pigment pelargonidin 3-sophoroside (C_27_H_31_O_15_) showing diagnostic ions at *m*/*z* 433.1080, 271.0595, 215.0695, 163.0596, and 127.0389, and peak 51 as pelargonidin 3-glucoside (C_21_H_20_O_10_) [37]. Those cyanidins possess antioxidant and anti-inflammatory activity [38].

#### 3.2.8. Citric Acid

Peaks 5 and 6 were assigned as citric acid and isocitric acid (C_6_H_8_O_7_) respectively.

#### 3.2.9. Carotenoids

Peaks 62 and 63 were tentatively identified as lutein (C_40_H_56_O_2_) and β-carotene (C_40_H_56_), respectively. In this study other carotene compounds were not detected, probably due to destruction by the pulping procedure. Lutein showed antioxidant and anti-inflammatory capacities, [39], and carotene showed anti-inflammatory, antioxidant and anticancer activity [40] and their presence in cocona fruits is in concordance with the medicinal.

### 3.3. Antioxidant Activity, Total Carotenes and Total Polyphenols Content

In this study the antioxidant capacities of the five ecotypes were assessed by the trapping of ABTS and DPPH and expressed as µmol Trolox/g dry matter (Table 4). In addition, total phenolic contents by the Folin and Ciocalteau’s method plus the total carotenes was assessed and correlated with the antioxidant capacities. Trapping of ABTS and DPPH radicals showed similar values in the different ecotypes and correlated with carotene and phenolic contents for the dry pulps. Strong correlation was found between total phenolics and DPPH antioxidant assays (r = 0.847, *p* < 0.0001). Moderated correlation was found between total carotenoids and ABTS antioxidant assays used (r = 0.640, *p* < 0.011). Ecotype UNT2 showed the highest DPPH• radical scavenging capacity in terms of content of Trolox equivalents (23.29 ± 1.07 µmol Trolox/g), but interestingly, in ABTS• radical scavenging activity the most powerful was the CD1 ecotype (25.67 ± 0.28 µmol Trolox/g).

### 3.4. Antihyperlipidemic Activities

Table 5 shows the variations in cholesterol, triglycerides, HDL and LDL in the different study groups. The highest cholesterol and triglyceride values correspond to the hypercholesteremic group that only received saline treatment (group II), with a statistically significant difference (*p* < 0.05) with the group control (group I) for the variables considered, thus corroborating the effectiveness of the model used. The increases obtained with Triton were 370 and 600 % for cholesterol and triglycerides, respectively. Several authors report similar elevations in the lipid profile of rodents treated with this agent. For instance, Harnafi et al. [41], obtained elevations of 270% for cholesterol and up to more than 1000% for triglycerides; while Khanna et al. [42], reported an elevation of cholesterol by 134%, these differences seem to depend on the strain of animals used.

When comparing the groups treated with the pulps, we noticed that samples which has received the CTR and SRN9 pulps showed the lowest cholesterol and triglyceride levels after treatment, however there is no clear correlations with the compounds detected and bioactivity showed by pulps. In agreement to the above, there isn’t a statistically significant difference (*p* < 0.05) between the cholesterol and triglyceride levels of the hypercholesterolemic group that received treatment with atorvastatin and the groups that received treatment with CTR and SRN9 pulps; which suggests that the treatment of the experimental animals with two pulps brings cholesterol levels closer to their basal values; this result is very important since it is an indicator of the effectiveness of the pulps tested. Regarding carotene compounds present in cocona fruits, it has been proved that diet with carotenes at a dose 80 mg/kg in rats decreased the lipid serum concentrations and its effect was comparable to that of simvastatin [43]. Regarding anthocyanins, also detected in cocona fruits, a juice from the fruit Aronia containing 106.8 mg cyanidin-3-glucoside equivalents/100 mL juice and 709.3 mg gallic acid equivalents/100 mL juice, showed an antihyperlipidemic effect in rats with hyperlipidemia and was proved to be valuable in reducing factors of cardiovascular risks [44]. Moreover, mulberry (*Morus alba* L.) lyophilized fruit administered to rats proved to have significant decrease in levels of serum and liver triglycerides, total cholesterol, serum low-density lipoprotein cholesterol, and a decrease in the atherogenic index, while the serum high-density lipoprotein cholesterol was significantly increased [45]. Several flavonoids and extracts rich in flavonoids were attributed anti hyperlipidemia activities; Pterocarpus marsupium heartwood and its flavonoid constituents, marsupsin, pterosupin, and liquiritigenin were able to effect a significant fall in serum cholesterol, LDL-cholesterol, and atherogenic index[46], also extracts full of glycosylated flavonoids from *Cardoncellus marioticus* showed antioxidant and antihyperlipidemic activities [47] moreover, using our same triton induced hyperlipidemia system extracts from the Mediterranean buckthorn, *Rhamnus alaternus* full of flavonoids decreased blood levels of cholesterol and triacylglycerols in hyperlipidemic rats (by 60% and 70%, respectively, at 200 mg CME/kg) [48]. In this study, all groups treated with the pulps showed a significant reduction in LDL-C (*p* < 0.05) with respect to the negative control group (group II), with the SRN9 group showing the best results. Despite the reduction in total cholesterol obtained with the different pulp treatments, we can observe that none of the treatments achieved lower values than the group treated with atorvastatin. In all the groups evaluated, an increase in HDL-C concentration was observed, the increase being significant in the groups that received CTR and SRN9 pulp (*p* < 0.05); the best result obtained was in the group that received SRN9 pulp after 96 hours of treatment. From the above mentioned we can say that SRN9 pulp, presents the best result of inhibition of the surfactant used (Triton WR-1339), by significantly decreasing the serum concentrations of cholesterol and triglycerides, corroborating the traditional use of this species and previous reports on its antihyperlipidemic activity [49,50]. This allows us to affirm that SRN9 pulp at the doses tested shows an antihyperlipidemic effect and could be an option in the management of people with high lipid levels. The presence of phenolic compounds, including flavonoids, could explain the hypolipidemic effect shown, presumably due to their proven antioxidant activity, as a result of a combination of their iron chelating and free radical scavenging properties [51,52]. During the experimental phase, no toxic effects were evidenced in the experimental specimens treated with NMA1; CD1; CTR; SRN9 and UNT2 pulps.

## 4. Conclusions

In this study, 70 compounds were detected in special Peruvian ecotypes of cocona fruits. Of those, six were spermidines, (peaks 1, 2, 25, 26, 38 and 39), thirteen were aminoacids, (peaks 3, 9, 11–13, 16, 17, 22–24), eighteen flavonoids (peaks 28, 30–32, 45, 46, 48–53, 56, 57, 61, and 64–66), twelve were phenolics (peaks 19, 21, 27, 29, 34, 35, 36, 42, 43, 44, 54, and 59), two carotenoids, (peak 62 and 63), eight were lipid derivatives (peaks 37, 55, 58, 60 and 67–70), one sugar (peak 47), four terpenes (peaks 33, 40, 41 and 47) two amides, (peaks 10 and 18), one aldehyde, (peak 15), and three saturated organic acids, (peaks 4, 5 and 20). Hypercholesterolemic mice administered with pulp of the ecotypes CTR and SRN9 showed the lowest cholesterol and triglyceride levels after treatment (126.74 ± 6.63; 102.11 ± 9.47; 58.16 ± 6.64; 61.05 ± 4.00 mg/dL, for cholesterol, triglycerides, HDL and LDL respectively, for the group treated with SRN9 pulp, and 130.09 ± 8.55; 108.51 ± 10.04; 57.30 ± 5.72; and 65.41 ± 7.68 mg/dL, for cholesterol, triglycerides, HDL and LDL respectively for the group treated with CTR pulp. Our study showed that the chemistry plus the bioactivity results obtained with five different ecotypes of Solanaceae Cocona fruits opens the door for the potential use of this plant to manage chronic diseases such as hyperlipidemia, especially for the SRN9 and CTR ecotypes, which presented better results in the antihyperlipidemic activity. The cultivation of these ecotypes using different conditions provides this common food plant with the ability to be a rich source of bioactive substances that can boost its consumption, not only as a fruit but also as natural medicine.

## Figures and Tables

**Table 1:** Proximal composition of cocona fruits NMA1, SRN9, CD1, CTR, UNT2 ecotypes.

Ecotypes	Humidity	Ashes	Total Lipids	Crude protein	Crude fiber	Carbohydrates
NMA1	91.85 ± 0.09 ^a^	0.75 ± 0.01 ^a^	0.65 ± 0.00 ^a^	1.08 ± 0.04 ^a^	1.68 ± 0.04 ^a^	3.99
CD1	86.64 ± 0.36 ^b^	1.24 ± 0.06 ^b^	0.88 ± 0.00 ^b^	1.93 ± 0.05 ^b^	5.03 ± 0.15 ^b^	4.28
CTR	92.82 ± 0.03 ^c^	0.71 ± 0.03 ^a^	0.45 ± 0.01 ^c^	1.09 ± 0.04 ^a^	1.03 ± 0.05 ^c^	3.9
SRN9	86.67 ± 0.12 ^b^	0.94 ± 0.02 ^c^	0.93 ± 0.01 ^d^	2.72 ± 0.04 ^c^	4.76 ± 0.17 ^b^	3.98
UNT2	93.52 ± 0.08 ^d^	0.79 ± 0.02 ^a^	0.19 ± 0.00 ^e^	1.64 ± 0.06 ^d^	0.76 ± 0.03 ^c^	3.1

Each value represents the means ± SEM of three replicates, *n* = 3, while different letters on the same column indicate significant difference using Tukey test at 0.05 level of significance (*p* < 0.05).

**Table 2:** Mineral content (mg/100 g fresh pulp) of cocona fruits NMA1, SRN9, CD1, CTR, UNT2 ecotypes.

Ecotypes	Fe	Zn	Mn	Cu	Mg	K	Na	Ca
NMA1	71.17 ± 2.69 ^a^	26.33 ± 0.95 ^a^	8.18 ± 0.30 ^a^	12.27 ± 0.52 ^a^	42.54 ± 0.93 ^a^	846.47 ± 19.85 ^a^	4.09 ± 0.13 ^a^	28.63 ± 0.86 ^a^
CD1	70.07 ± 2.65 ^a^	67.05 ± 1.56 ^b^	34.35 ± 1.16 ^b^	41.22 ± 0.73 ^b^	164.88 ± 6.55 ^b^	2382.24 ± 29.95 ^b^	6.87 ± 0.13 ^b^	70.07 ± 1.62 ^b^
CTR	40.70 ± 1.82 ^b^	17.83 ± 0.84 ^c^	7.85 ± 0.15 ^ac^	11.42 ± 0.42 ^ac^	38.56 ± 0.69 ^ac^	638.10 ± 6.28 ^c^	3.57 ± 0.09 ^c^	17.85 ± 0.46 ^c^
SRN9	58.09 ± 1.15 ^c^	45.93 ± 2.19 ^d^	9.46 ± 0.08 ^d^	14.86 ± 0.65 ^d^	91.87 ± 1.54 ^d^	2004.88 ± 33.34 ^d^	6.76 ± 0.18 ^b^	58.09 ± 1.05 ^d^
UNT2	52.02 ± 1.17 ^d^	18.85 ± 0.90 ^c^	8.45 ± 0.21 ^acd^	10.40 ± 0.30 ^c^	41.60 ± 0.84 ^ac^	570.83 ± 12.13 ^e^	3.25 ± 0.04 ^c^	41.60 ± 0.72 ^e^

Each value represents the means ± SEM of three replicates, *n* = 3, and different letters on the same column indicate significant difference using Tukey test at 0.05 level of significance (*p* < 0.05).

**Table 3:** High resolution UHPLC-PDA-ESI-OT-MS identification of metabolites in fractions of cocona fruits (a–e): NMA1, SRN9, CD1, CTR, UNT2 ecotypes, respectively.

Peak#	Retention Time (min.)	UV Max	Tentative Identification	Molecular Formula	Theoretical Mass (*m*/*z*)	Measured Mass [M-H]^−^ or [M+H]^+^ (*m*/*z*)	Accuracy (ppm)	MS^n^ Ions	Ecotype
1	1.25	-	Spermine	C_10_H_26_N_4_	203.22302	203.2229	−2.448	129.1385, 112.1122, 84.0812, 73.0813	a–e
2	1.33	-	Spermidine	C_7_H_19_N_3_	146.16517	146.1651	−1.96	129.1385, 112.1122, 84.0812, 73.0813	a–e
3	1.35	-	Histamine	C_5_H_9_N_3_	112.08692	112.0872	0.79	95.0606, 83.0608, 68.0500, 55.0549	a–e
4	1.69	-	Citric acid	C_6_H_8_O_7_	191.01944	191.01863	4.24	129.1385	c–e
5	1.75	-	Isocitric acid	C_6_H_8_O_7_	191.01944	191.01947	3.25	111.00794	d–e
6	1.97	-	Asparagine	C_4_H_8_N_2_O_3_	131.0449	131.0454	3.81	114.0187, 113.0337, 95.0251, 88.0394 70.0288	a–b
7	2.29	-	Arginine	C_6_H_14_N_4_O_2_	175.1181	175.1188	3.99	158.0920, 140.0702, 130.0972, 116.0706 97.0661 70.0656	c
8	2.43	-	Pyroglutamic acid	C_5_H_7_NO_3_	130.0493	130.0499	4.61	102.0251, 84.0448, 56.0551,	a–b
9	3.01	-	Nicotinamide	C_6_H_6_N_2_O	123.0550	123.0553	2.43	106.0289, 96.0446, 80.0499, 53.0391	c–e
10	3.15	-	N-phenyl ethyl amide	C_8_H_10_N	120.08810	120.08080	2.42	85.02876	a–b
11	4.1	-	N-Fructosyl isoleucine	C_12_H_23_NO_7_	294.1541	294.1544	1.01	276.1436, 258.1332, 230.1383, 212.1278 161.0681, 144.1017, 132.1017, 86.0968	c–e
12	4.32	-	Norleucine	C_6_H_13_NO_2_	132.1016	132.1019	2.27	105.0696, 86.0968, 69.0704	c–e
13	5.21	-	Tyrosine	C_9_H_11_NO_3_	188.0210	188.0211	0.53	165.0554, 147.0438, 136.0755, 123.0441 105.0337	a–e
14	7.03	-	Adenosine	C_10_H_13_N_5_O_4_	268.1037	268.1039	0.74	178.0730, 136.0616, 57.0341	c–e
15	8.12	-	Phenylacetaldehyde	C_8_H_8_O	121.0642	121.0649	5.48	103.0544, 93.0702, 91.0546, 77.0387 53.0392	c–e
16	8.53	-	Guanosine	C_10_H_13_N_5_O_5_	284.0983	284.0988	1.75	152.0564, 133.0494, 121.0647, 95.0609	c–e
17	9.62	-	Phenylalanine	C_9_H_11_NO_2_	166.0859	166.0861	1.20	149.0594, 131.0491, 120.0808, 103.0543 93.0703	c–e
18	10.23	-	Aminobutyl benzamide	C_11_H_16_N_2_O	193.1332	193.1336	2.07	176.1067, 134.0599, 105.0337, 72.0813	a–b
19	10.34	290–335	Chlorogenic acid	C_8_H_14_O_4_^−^	353.0863	353.0882	4.21	191.05574, 707.18678	b–c
20	10.5	208	Quinic acid	C_7_H_11_O_6_^−^	191.0550	191.0557	2.34	135.04477, 85.02844	b–e
21	10.88	235	3-O-diglucosyl-4-methoxy-3-hydroxybenzoic acid	C_20_H_27_O_14_^−^	491.1395	491.1412	3.46		a–d
22	11.25	-	Pantothenic acid	C_9_H_17_NO_5_	220.1173	220.1179	2.72	202.1069, 184.0965, 160.0965, 142.0860 124.0756	a–d
23	11.43	-	Tryptophan	C_11_H_12_N_2_O_2_	205.0961	205.0968	3.41	188.0701, 170.0596, 159.0914, 146.0597 132.0804 118.0650	a–b
24	11.75	-	Tryptophol	C_10_H_11_NO	144.0802 [M- H_2_O+H]	144.0807	3.47	128.0491, 117.0699, 103.0506, 91.0547	a–b
25	11.97	330	N-Caffeoyl-N-(dihydrocaffeoyl)spermidine	C_25_H_33_N_3_O_6_	472.2439	472.2441	0.42	455.2163, 310.2118, 293.1852, 222.1120 163.0386, 72.0813	a
26	12.03	330	N-Caffeoyl-N-(dihydrocaffeoyl)spermidine	C_26_H_37_N_3_O_6_	488.2751	488.2756	1.02	471.2478, 324.2273, 293.1844, 236.1275 222.1119, 165.0542	a–b
27	12.02	325	3-O-Diglucosyl-4-methoxy-3-hydroxybenzoic acid	C_20_H_27_O_14_^−^	491.13953	491.14124	3.46		a–d
28	12.24	254–354	Rutin	C_27_H_30_O_16_	609.14702	609.14709	0.11	463.0920, 343.0465, 301.0254, 300.0280, 271.0252 178.9982, 151.0031	b–c
29	12.46	240	Apiosyl-(1→6)-glucosyl 4-hydroxybenzoate	C_18_H_24_O_12_	431.0980	431.0983	0.46	431.1196, 299.0768, 281.0679, 137.0237 93.0336	b–d
30	14.03	280	Naringenin-5,7-di-O-D-glucopyranoside	C_27_H_31_O_15_^−^	595.16575	595.16772	3.32	271.06152, 153.01845, 147.04482, 119.05661	c–e
31	14.21	280	Genistein 5-O-glucoside	C_21_H_20_O_10_	431.0980	431.0984 433.1123	0.46	414.3355, 271.0595, 269.0390, 253.0485, 215.0698 146.0598, 127.0389, 85.0288	d–e
32	15.02	254–354	Isoquercitrin	C_21_H_20_O_12_	463.1022	463.1027	1.08	300.0280, 271.0251, 255.0301, 178.9982 151.0032	c–e
33	15.21	-	Spirosol-5-en-3-ol, 3-O-[Rhamnosyl-(1→2)- glucosyl-(1→3)]-galactoside	C_45_H_73_NO_16_	884.4982	884.4987 928.4909 [M+FA-H]	0.56	722.4060, 576.3906, 414.3356	b–e
34	15.24	329	1-*O*-Sinapoyl-glucoside	C_17_H_22_O_10_	385.1142	385.1147	1.29	247.0612, 223.0611, 205.0504, 190.0269 164.0704, 119.0342,	b
35	15.36	280	Protocatechuic acid 5-O-[apiofuranosyl-(1→6)-glucopyranoside]	C_19_H_26_O_13_	461.1301	461.1302	0.21	329.0872, 167.0344, 152.0108, 123.0443 108.0208	b–c
36	15.57	325	4-O-(3′-O-Glucopyranosyl)-caffeoyl quinic acid	C_22_H_28_O_14_	515.1401	515.1407	1.16	395.0990, 353.0876, 191.0557, 179.0344 161.0238, 135.0444	a–e
37	15.87	-	Phytosphingosine	C_18_H_39_NO_3_	318.2990	318.2995	1.57	300.2890, 282.2785, 270.2785, 60.0450	a, b, d, e
38	16.23	280	N,N″-Bis[3-(4-hydroxy-3-methoxyphenyl)propanoyl] spermidine	C_27_H_39_N_3_O_6_	502.2902	502.2907	0.99	485.2633, 307.1996, 236.1275, 179.0698 137.0594	a–b
39	16.73	325	N,N,N-tris(dihydrocaffeoyl) spermidine	C_34_H_43_O_9_N_3_	638.3059	638.3062	0.46	474.2588, 456.2484, 293.1852, 222.1120 165.0543, 123.0439	a–b
40	17.23	-	Spirosol-5-en-3-ol, O-[Rhamnosyl-(1→2)-[xylosyl- (1→2)-rhamnosyl-(1→4)]-galactoside	C_50_H_81_NO_19_	1000.5450	1000.5456	0.59	868.4970, 722.4737, 576.3879, 414.3358	a–b
41	17.55	-	Cholest-5-ene-3,16,22,26-tetrol, 3-O-[Rhamnosyl- (1→4)-[rhamnosyl-(1→2)]glucoside], 26-O- glucoside	C_51_H_86_O_22_	1051.5660	1051.5665	0.47	1049.5541, 903.4961, 757.4382, 595.3851 433.3324	a–b
42	17.67	330	3-O-Feruloylquinic acid	C_17_H_20_O_9_	367.1032	367.1039	1.90	191.0558, 173.0451, 134.0366, 111.0443 93.0336,	a–b
43	18.05	329	2-*O*-Sinapoyl-glucoside	C_17_H_22_O_10_	385.1141	385.1147	1.55	247.0612, 223.0611, 205.0504, 190.0269 164.0704, 119.0342	a–b
44	19.01	330	Syringaresinol 4-gentiobioside	C_34_H_46_O_18_	787.2676	787.2678 [M+FA-H]	0.25	417.1560, 402.1323, 387.1069, 371.1494 356.1233, 181.0502, 166.0266	a–e
45	19.53	280	Naringenin 7- O-rutinoside	C_27_H_31_O_14_^−^	579.17083	579.17291	3.59	271.06152, 151.00319	a–b
46	19.72	254–354	Quercetin 3-galactoside	C_12_H_19_O_5_^−^	243.12270	463.08893	3.94	350.20898, 301.02795, 151.00310	a–e
47	20.45	-	Spirosol-5-en-3-ol, 3-O-[Rhamnosyl-(1→2)-[rhamnosyl-(1→4)]-glucoside]	C_45_H_73_NO_15_	868.5031	868.5035	0.46	722.4411, 576.3845, 414.3357	a, b, d, e
48	22.32	280	Biochanin A 7-O-rutinoside	C_28_H_32_O_14_	593.1852	593.1859	1.18	447.1269, 327.0856, 285.0750, 153.0191	a, b
49	22.51	280	Genistin	C_21_H_20_O_10_	431.0982	433.1123 431.0984	0.69	414.3355, 271.0595, 253.0485, 215.0698 146.0598, 127.0389, 85.0288,	a–e
50	23.35	255–340	3,5-Dihydroxy-4′,7-dimethoxyflavone 3-O-[Rhamnosyl-(1→2)-glucoside (Pectolarin)	C_29_H_34_O_15_	623.1961	623.1963	0.32	477.1342, 315.0815, 300.0622, 284.0679	a, b, d, e
51	24.93	281	Naringenin-7-O-glucoside	C_21_H_22_O_10_	435.1279	435.1282 433.1117	0.95	313.0506, 271.0617, 193.0138, 151.0032 119.0405	d–e
52	25.5	520	Pelargonidin 3-O-sophoroside	C_27_H_31_O_15_	595.1652	595.1656	0.67	433.1080, 271.0595, 215.0695, 163.0596 127.0389	a–b
53	26.1	520	Pelargonidin 3-O-glucoside	C_21_H_20_O_10_	433.1121	433.1126	1.15	311.0556, 269.0460, 163.0031	a–b
54	26.5	325	Methyl chlorogenate	C_17_H_20_O_9_	367.1031	367.1035	1.08	191.0556, 179.0344, 161.0237, 135.0443 93.0335	a–d
55	26.7	-	Peak 55, 1-Hexadecanoyl-sn-glycero-3-Phosphoethanolamine	C_21_H_44_NO_7_P	454.2920	454.2923 452.2786	0.66	255.2630, 214.0482, 214.0284, 140.0111	c–e
56	27.0	280	Naringenin-5-O-glucoside	C_21_H_22_O_10_	433.1132	433.1138	1.38	313.0551, 271.0613, 151.0030, 119.0493 93.00335	a–e
57	27.1	280	Eriodictyol-7-O-glucoside	C_21_H_22_O_10_	449.1080	449.1083	0.66	287.0567, 205.0144, 175.0033, 151.0032 135.0445, 125.0237	a–e
58	27.2	-	1-Hexadecanoyl-sn-glycero-3-phospho-(1′-myo-inositol)	C_25_H_49_O_12_P	573.3030	573.3034 571.2089	0.69	391.2256, 333.0594, 315.0487, 255.2329 241.0118, 152.9951	a–e
59	27.3	330	Syringaresinol-glucoside	C_28_H_36_O_13_	579.2080	579.2084	0.89	417.1557, 402.1320, 387.1085, 223.0616 181.0501, 166.0265	c
60	27.5	-	1-(9Z-Octadecenoyl)-sn-glycero-3-phospho-(1′-myo-inositol)	C_27_H_51_O_12_P	599.3189	599.3192 597.3046	0.50	333.0594, 315.0488, 281.2487, 259.0223 241.0118	d–e
61	27.7	254–354	Quercetin 3-O-malonylglucoside	C_24_H_22_O_15_	579.2080	579.2084	0.69	463.0888, 300.0280, 271.0252, 255.0301 178.9981, 151.0032	a–e
62	27.9	450	Lutein	C_40_H_56_O_2_	568.4280	568.4282		124.08689, 145.0845, 105.08564, 335.12485	a–e
63	28.0	450	β-carotene	C_40_H_56_	536.4379	536.4382		337.09189, 476.17609	a–e
64	28.1	280	Naringenin	C_15_H_12_O_5_	271.06010	271.06155	5.36	153.01832, 147.04453, 119.05632	e
65	28.3	280	Phloretin	C_15_H_14_O_5_	349.18569	349.18723	4.03	229.0871, 179.0347, 167.0347, 125.0237	e
66	28.4	254–354	Cirsimarin	C_23_H_24_O_11_	475.12668	475.12524	4.36	315.07642	c–e
67	28.9	-	1-(9Z-Octadecenoyl)-sn-glycero-3-phosphoethanolamine	C_23_H_46_NO_7_P	480.3080	480.3085 478.2937	0.83	281.2486, 214.0482, 196.0386, 152.9950 140.0110	a–b
68	29.2	-	1-hexadecanoyl-sn-glycero-3-phosphocholine	C_24_H_50_NO_7_P	496.3391	496.3396	1.00	313.2128, 184.0721, 124.9998, 104.1072 86.0968	a–b
69	29.7	215	1-(9Z-Octadecenoyl)-sn-glycero-3-phosphocholine	C_26_H_52_NO_7_P	566.3419	566.3422 [M+FA-H] 522.3555	0.56	504.3450, 445.2715, 339.2892, 240.1001 199.0370, 187.0732, 124.9988	c–e
70	30.2	210	1-Oleoyl-2-palmitoyl-sn-glycero-3-phosphocholine	C_42_H_82_NO_8_P	760.5832	760.5839 758.542	0.92	184.0730, 125.9997, 104.1071, 86.0964	a–b

**Table 4:** Antioxidant activity (DPPH, ABTS), total phenolic content and total carotenoid contents of cocona fruits pulps NMA1, SRN9, CD1, CTR, UNT2 ecotypes.

Ecotypes	DPPH (µmol Trolox/g)	ABTS (µmol Trolox/g)	Total Phenolics (mg GAE/g)	Total Carotenoids (μg β-carotene)/g)
NMA1	19.88 ± 0.34 ^a^	19.70 ± 0.81 ^a^	32.68 ± 1.33 ^a^	101.22 ± 4.47 ^a^
CD1	18.37 ± 0.24 ^b^	25.67 ± 0.28 ^b^	28.03 ± 0.90 ^b^	122.65 ± 4.24 ^b^
CTR	21.92 ± 0.53 ^c^	21.98 ± 0.90 ^ac^	35.79 ± 0.84 ^ac^	85.13 ± 1.81 ^c^
SRN9	18.21 ± 0.24 ^bd^	23.97 ± 1.12 ^bc^	27.86 ± 0.81 ^b^	92.12 ± 3.64 ^ac^
UNT2	19.15 ± 0.84 ^abd^	20.25 ± 0.79 ^ac^	34.26 ± 1.32 ^ac^	58.81 ± 0.46 ^ab^

Each value represents the means ± SEM of three replicates, *n* = 3, while different letters on the same column indicate significant difference using Tukey test at 0.05 level of significance (*p* < 0.05).

**Table 5:** Effect of cocona fruit pulps NMA1, CD1, CTR, SRN9, UNT2 in serum biochemical parameters on Triton induced hyperlipidemic rat.

Groups	Cholesterol	Triglyceride	HDL	LDL
Group I (control)	78.9 ± 5.90	72.28 ± 3.98	50.97 ± 4.02	27.97 ± 5.26
Group II hypercholesterolemic, saline treated	378.17 ± 6.27 ^a^	461.65 ± 8.82 ^a^	42.68 ± 3.14	225.64 ± 12.16 ^a^
Group III hypercholesterolemic, atorvastatin treated	118.41 ± 10.05 ^b^	93.90 ± 11.23 ^b^	60.13 ± 5.08 ^b^	50.79 ± 4.46 ^b^
Group IV hypercholesterolemic, NMA1 pulp treated	302.76 ± 17.87 ^a^	268.90 ± 7.92 ^a^	47.44 ± 5.06	145.33 ± 9.56 ^a^
Group V hypercholesterolemic, CD1 pulp treated	287.28 ± 10.03 ^a^	259.63 ± 13.24 ^a^	48.12 ± 8.07	139.06 ± 8.07 ^a^
Group VI hypercholesterolemic, CTR pulp treated	130.09 ± 8.55 ^b^	108.51 ± 10.04 ^b^	57.30 ± 5.72 ^b^	65.41 ± 7.68 ^b^
Group VII hypercholesterolemic, SRN9 pulp treated	126.74 ± 6.63 ^b^	102.11 ± 9.47 ^b^	58.16 ± 6.64 ^b^	61.05 ± 4.00 ^b^
Group VIII hypercholesterolemic, UNT2 pulp treated	338.81 ± 15.95 ^ac^	299.86 ± 17.81 ^ac^	45.56 ± 7.46 ^c^	165.85 ± 7.42 ^ac^

Values represent the mean ± SD for observations made on six rats in each group. Units: milligrams per deciliter. ^a^ Statistically significant difference (*p* < 0.05) when compared with group I values, ^b^ Statistically significant difference (*p* < 0.05) when compared with group II values, ^c^ Statistically significant difference (*p* < 0.05) when compared with group III values.

## Data Availability

The data presented in this study are available in this article.
